# Case Report: Two Cases of Chemotherapy Refractory Metastatic Penile Squamous Cell Carcinoma With Extreme Durable Response to Pembrolizumab

**DOI:** 10.3389/fonc.2020.615298

**Published:** 2020-12-23

**Authors:** Jad Chahoud, William Paul Skelton, Philippe E. Spiess, Christine Walko, Jasreman Dhillon, Kenneth L. Gage, Peter A. S. Johnstone, Rohit K. Jain

**Affiliations:** ^1^Department of Genitourinary Oncology, H. Lee Moffitt Cancer Center and Research Institute, University of South Florida, Tampa, FL, United States; ^2^Division of Medical Oncology, H. Lee Moffitt Cancer Center and Research Institute, University of South Florida, Tampa, FL, United States; ^3^Division of Individualized Cancer Management, Personalized Medicine, H. Lee Moffitt Cancer Center and Research Institute, University of South Florida, Tampa, FL, United States; ^4^Department of Anatomic Pathology, H. Lee Moffitt Cancer Center and Research Institute, University of South Florida, Tampa, FL, United States; ^5^Department of Diagnostic Imaging and Interventional Radiology, H. Lee Moffitt Cancer Center and Research Institute, University of South Florida, Tampa, FL, United States; ^6^Departments of Radiation Oncology, H. Lee Moffitt Cancer Center and Research Institute, University of South Florida, Tampa, FL, United States

**Keywords:** immunotherapy, penile cancer, durable response, pembrolizumab, metastatic

## Abstract

**Background:**

Penile squamous cell carcinoma (PSCC) is a rare malignancy, and those patients with metastatic disease have limited treatment options. Treatment is largely comprised of platinum-based chemotherapy; however, patients progressing after initial chemotherapy have a median overall survival (OS) of less than 6 months. Based on a high percentage of PD-L1 expression in patients with PSCC, and its biological similarities to other squamous cell carcinomas, we present two patient cases treated with pembrolizumab with extraordinary durable treatment response far beyond treatment with standard therapy.

**Main Body:**

The first patient is a 64 year old male with PSCC who was treated with neoadjuvant chemotherapy, partial penectomy, and adjuvant radiation prior to developing metastatic disease. He had a high TMB (14 mutations/Mb) and was started on pembrolizumab with a complete response, which has been maintained for 38 months. The second patient is an 85 year old male with PSCC who was treated with partial penectomy and adjuvant chemotherapy and radiation prior to developing metastatic disease. He had positive PD-L1 expression CPS 130) and was started on pembrolizumab with a partial response, which has been maintained for 18 months after starting treatment.

**Conclusions:**

These two cases of extreme durable response with pembrolizumab (with molecular data including TMB and PD-L1 status) represent a significant clinical benefit in this patient population. With limited treatment options that result in a median OS of less than 6 months, along with the toxicity profile of chemotherapy which may not be tolerated in elderly patients with comorbidities, this survival benefit with pembrolizumab, along with advances in tumor sequencing and clinical trials shows that there is a potentially significant benefit with novel therapies in this patient population.

## Introduction

Penile squamous cell carcinoma (PSCC) is a very rare malignancy, accounting for 0.12% of malignancies in the United States in 2020 but 0.73% of cancer deaths (1). Nevertheless, in 2018 around 35,000 new cases of PSCC were reported (2, 3). Between 30% and 50% of penile cancers are related to human papillomavirus (HPV), particularly subtypes 6, 16, and 18 (4, 5). Despite HPV positive status being an increased risk for the development of penile carcinoma, those with penile carcinoma with a positive HPV status have improved outcomes (6). The treatment of PSCC is largely dependent on the stage of the tumor, with initial management of localized disease usually being surgical resection (7). Lymphatic involvement has been shown to be a strong prognostic factor for survival, as patients with no involved lymph nodes have 5-year survival of 96%, with survival decreasing incrementally with more involved nodal groups (80% for N1, 66% with N2, and 37% with N3) (8). Unfortunately, patients with locally advanced penile carcinoma are likely to recur, as 70% of patients will have recurrent disease despite neoadjuvant chemotherapy with combination chemotherapy with cisplatin, paclitaxel and ifosfamide followed by surgical lymph node dissection (9). Also, for patients presenting with visceral or metastatic disease, treatment options are largely based on performance status (10) and outcomes are generally dismal and estimated to < 5 months. For those with adequate performance status, potential regimens include different chemotherapy regimens including combinations of paclitaxel/ifosfamide/cisplatin (10), cisplatin/fluorouracil (11), cisplatin/irinotecan (12), paclitaxel/carboplatin (13), and monotherapy with the EGFR inhibitor cetuximab in some patients. Paclitaxel monotherapy showed a median PFS of 11 weeks (95% CI, 7–30) and median OS of 23 weeks (95% CI, 20–48), and was well tolerated with the most common grade 3/4 side effect neutropenia in 7 patients (28%) (14). Unfortunately, patients who progress following initial chemotherapy have a median overall survival (OS) of less than 6 months (15) with no currently accepted standard of care. Given the poor survival following progression as well as the lack of treatment options in this patient population, further investigation of novel therapeutical approaches is necessary.

Immune check point inhibitors (ICB) have changed the treatment landscape in many solid tumors over the past decade with multiple FDA drug approval, specifically in other advanced solid tumors with squamous cell histology. The lack of durable response to conventional chemotherapy including taxanes and platinums has led to numerous investigative efforts exploring the role of immunotherapy. Cemiplimab, approved for the treatment of patients with advanced cutaneous squamous-cell carcinoma, induced a response in approximately half of patients (16). Also, for patients with metastatic or recurrent squamous cell carcinoma of the head and neck following progression on platinum-based therapy, nivolumab is approved (17). The molecular and viral similarities between PSCC and these SCCs can be the rationale for investigation of these therapies in patients with advanced PSCCs (18–20). In addition, 62% of patients with metastatic penile cancer were shown to be positive (≥5%) for PD-L1 expression, along with a strong correlation of PD-L1 expression in primary and metastatic samples (21). It has also been shown that 32% to 62% of squamous cell penile cancers test positive for PD-L1 expression, and thus may have a role as a predictive biomarker of response to immunotherapy (21–25).

Another important potential marker of ICB response in solid cancers is the tumor mutational burden (TMB). A recent paradigm-shifting study has resulted in the FDA approving pembrolizumab in patients with unresectable or metastatic solid tumors which harbor a high tumor mutational burden (TMB) in those who have either progressed on prior therapy or have no alternative treatment options, and is of note an approval despite the site of the primary tumor (26).

Based on the above rationale, we present 2 cases of patients with metastatic penile carcinoma with high tumor mutational burden and PDL-1 expression in penile tumor tissue who were treated with pembrolizumab with excellent response.

## Case Presentation A (Patient 1)

**Figure 1:**
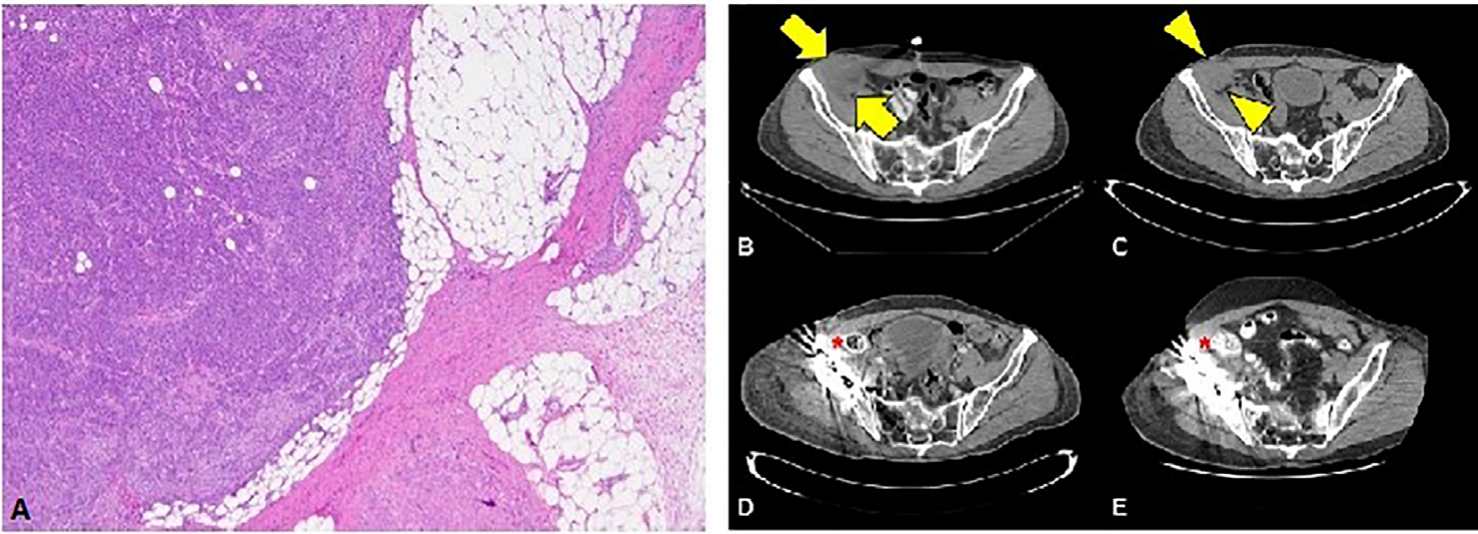
**(A)** Squamous cell carcinoma with perinodal extension into fat (4X magnification). **(B)** Cross section through the CT portion of the PET/CT prior to the initiation of immunotherapy. The largest lymph node measures 5.6 x 4.3 cm transverse (arrows). **(C)** Noncontrast CT of the pelvis obtained approximately 1 month after initiation of immunotherapy reveals decreasing lesion size (arrowheads) now measuring 4.2 x 3.8 cm (arrowheads). Subsequent contrast enhanced CTs at 3 months **(D)** and 36 months **(E)** after initiation of immunotherapy show no evidence of residual nodal disease.

The first patient is a 64-year-old male with no past medical history who was originally diagnosed with moderate to poorly differentiated keratinizing squamous cell carcinoma of the penis in 2015. He underwent 4 cycles of neoadjuvant chemotherapy with TIP (paclitaxel, ifosfamide, and cisplatin) and then underwent partial penectomy with lymph node dissection and was found to have bilateral inguinal lymph node metastases with extranodal extension, along with a positive right obturator pelvic lymph node metastasis (stage IV TxN3M0) (**Figure 1A**). HPV status was unknown. Thereafter, he received adjuvant external beam radiation therapy (EBRT) with 57.4 Gy in 28 fractions concurrently with weekly cisplatin. Surveillance imaging May of 2017 revealed increasing hypermetabolic right pelvic adenopathy (biopsy-proven, largest lymph node measuring 5.6 × 4.3 cm) along with new bone metastasis in the right anterior acetabulum (**Figure 1B**). Foundation One molecular testing of an inguinal lymph node revealed 7 alterations in clinically relevant genes, including PTCH1 S1203fs*52 (variant allele frequency [VAF] 19.2%), EP300 N419fs*12 (VAF 20.3%), FAT1 S1669* (VAF 33.1%), HSD3B1 G171R (VAF 1.2%), MLL2 L4921fs*74 (VAF 21.9%), MLL2 P2354fs*30 (VAF 22.9%), and QKI K134fs*14 (VAF 24.4%). There were also 17 variants of unknown significance. The microsatellite status could not be assessed but the tumor mutational burden (TMB) was high at 14 mutations/megabase, (**Table 1**). His PD-L1 status was not assessed. Given the fact that he had refractory disease to platinum-based chemotherapy and chemoradiation, with the noted high TMB and the high prevalence of PD-L1 expression in penile SCC, he was started on pembrolizumab 7/2017 and subsequently underwent a wide resection hemipelvectomy with acetabular reconstruction and total hip arthroplasty the following month. Imaging 3 months after starting pembrolizumab showed complete resolution of lymph node metastasis with no evidence of metastatic disease (**Figures 1C, D**). Surveillance scans have continued every 2 months to show no evidence of metastatic disease 38 months after starting pembrolizumab with last follow-up 8/2020 (**Figure 1E**). He has tolerated the pembrolizumab well with no grade 3 or higher AEs, his only immune related adverse event being grade 2 hypothyroidism for which he was started on levothyroxine with normalization of thyroid function.

**Table 1:** Clinicopathologic characteristic of patients with metastatic prostate cancer treated with immunotherapy.

	Response	Duration of Follow up	Adverse Events (AEs)	HPV status	PDL-1 status	MSI status	TMB	Molecular mutations
Patient 1	CR	38 months	Hypothyroidism	Unknown	Unknown	Ambiguous	High (14 mutations/Mb)	PTCH1 S1203fs*52 (VAF 19.2%) EP300 N419fs*12 (VAF 20.3%) FAT1 S1669* (VAF 33.1%) HSD3B1 G171R (VAF 1.2%) MLL2 L4921fs*74 (VAF 21.9%) MLL2 P2354fs*30 (VAF 22.9%) QKI K134fs*14 (VAF 24.4%)
Patient 2	PR	18 months	N/A	Negative	Positive (CPS 130)	Stable	Low (3 mutations/Mb)	MYD88 L265P (VAF 1.5%) NFE2L2 W24R (VAF 36.4%) SMARCA4 M1233I (VAF 8.9%) TERT promoter 146C>T (VAF 18.7%) TP53 R280G (VAF 18.3%)

## Case Presentation B (Patient 2)

**Figure 2:**
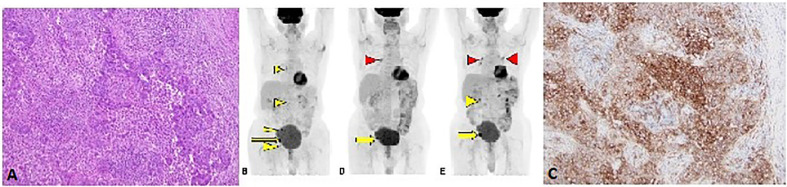
**(A)** H&E stain from lymph node metastasis (10X magnification). **(B)** F18-FDG PET/CT Coronal MIP projection obtained prior to initiation of immunotherapy. The yellow arrow indicates the dominant lesion, while the yellow arrowheads indicate additional nontarget and/or suspicious lesions. **(C)** PD-L1 stain showing strong membranous staining in the tumor cells (10X magnification). Follow-up PET/CT **(D)** approximately 3 months after initiation of immunotherapy reveals resolution or near resolution of the nontarget lesions with decrease in dominant lesion. There is new focus of radiotracer accumulation in the right hilum, which is of unclear clinical significance (red arrowhead). The six months follow-up PET/CT **(E)** reveals grossly symmetric FDG avid hilar regions (red arrowheads) suggesting possible development of sarcoidosis of sarcoid-like reaction, an uncommon but reported finding in immunotherapy (27). There has been interval enlargement of the dominant lesion and re-emergence of some suspicious lymph nodes (yellow arrow and arrowhead).

The second patient is an 85-year-old male with no past medical history who was diagnosed with moderately differentiated squamous cell carcinoma of the penis in 2017. He underwent partial penectomy and glansectomy and shortly after the procedure was found to have a 1.5-cm palpable right inguinal lymph node, for which biopsy confirmed metastatic squamous cell carcinoma (no extranodal extension or lymphovascular/perineural invasion). HPV testing was negative (subtypes 6, 11, 16, 18, 31, 33, 45, and 58 were not detected). He underwent bilateral inguinal lymph node dissection, which showed a positive right inguinal lymph node with extranodal extension (**Figure 2A**). Final surgical staging stage IV TxpN3M0. He underwent four cycles of adjuvant chemotherapy with cisplatin and 5-FU. Following chemotherapy, he underwent adjuvant radiation to the bilateral inguinal lymph nodes (45 Gy in 25 fractions) and a right inguinal lymph node boost with 2160 cGy in 12 fractions. He was followed with surveillance scans and 5 months following completion of radiation, was found to have avid lymphadenopathy in the subcarinal region and right hilum of the chest, along with progression of adenopathy in the retroperitoneum and pelvis (**Figure 2B**), of which biopsy confirmed chemotherapy and radiation refractory metastatic squamous cell carcinoma. Analysis of his FoundationOne CDx molecular testing of an inguinal lymph node revealed 5 mutations, including MYD88 L265P (VAF 1.5%), NFE2L2 W24R (VAF 36.4%), SMARCA4 M1233I (VAF 8.9%), TERT promoter 146C>T (VAF 18.7%), and TP53 R280G (VAF 18.3%). There were also 6 variants of unknown significance. The microsatellite status was stable and the tumor mutational burden was low at 3 mutations/megabase. His PD-L1 was markedly positive with a CPS (combined positive score) of 130 (**Figure 2C**). Based on the tumor strongly positive’s PD-L1 status, he was started on pembrolizumab 3/2019 and surveillance scans 3 months thereafter showed marked improvement in adenopathy (**Figure 2D**). Imaging 10 months after starting pembrolizumab showed some progression of adenopathy but overall his disease has been controlled since starting pembrolizumab 18 months ago (**Figure 2E**), consistent with partial response (PR) and disease control from stabilization of an otherwise deadly tumor within months. This 85-year-old gentleman tolerated pembrolizumab without any reported AEs and continued to have clinical benefit with significant improvement in daily activity level in comparison to the time of recurrence diagnosis.

## Discussion

We present two cases of patients treated with pembrolizumab for refractory metastatic penile cancer with disease progression after multiple lines of chemotherapy and radiation therapy with excellent response and very durable clinical benefit. The rationale to treat these patients with this rare cancer with single agent ICB was noting a high TMB in one patient and markedly positive PDL-1 expression on IHC staining in the other patient. As Wang et al. showed, patients progressing following initial chemotherapy have a median OS of less than 6 months (15). Many established treatment options are toxic and may not be tolerated well in elderly patients with comorbidities or decreased performance status. This report highlights the potential clinical benefit of testing ICB response and therapy with single agent ICB in patients with PSCC when enrollment on clinical trials is not possible. A recent Phase II trial examining the combination of nivolumab with ipilimumab enrolled 6/56 patients with penile carcinoma, where 2/6 had stable disease, 3/6 progressive disease, and 1 was not evaluated at a median follow up of 9.9 months (28). The TMB, PDL-1 expression and other markers of response to therapy from the patients tissue on this trial were not presented as part of the preliminary data presentation. In contrast, the two patients that we report on had markers of immunotherapy response have had tumor response to therapy and excellent clinical benefit 38 and 18 months following initiation of treatment with pembrolizumab. This is a very significant clinical impact, as with standard therapies these two patients would survive for a very short time and with significant toxicity-related side effects as a result of treatment).

The recent FDA-approval for use of pembrolizumab in solid tumors harboring a high TMB portends a potential promising outcome for this patient cohort (26) and thus all patients with refractory PSCC should receive genomic testing for TMB. It has also been shown that 21% of penile cancer patients had an increased TMB of more than 10 mutations per Mb, thereby portending a potential increased response to immunotherapy (19). There are currently two phase II trials examining immunotherapy with pembrolizumab (NCT028307042) and avelumab (NCT03391479) in patients with advanced penile cancer, the results of which are eagerly anticipated. The optimal biomarker for directing immune checkpoint inhibitor therapy is unclear and may be histology specific in certain circumstances, though evolving research across solid tumors has elucidated the value of TMB and PD-L1 positivity, as well as more specific measurements of immune cell infiltration into the tumor and related microenvironment (29). Mutation burden has been shown to be a surrogate marker for neoantigens which have been associated with response to immune checkpoint inhibitors, including ipilimumab in melanoma and pembrolizumab in lung cancer. Based on these observations across malignancies and the results of the KEYNOTE-158 trial, pembrolizumab was granted accelerated approval for the treatment of patients with unresectable or metastatic solid tumors with a TMB ≥ 10 who have progressed on prior therapy (26). Recent data has also shown that immunotherapy combined with targeted therapeutics may have a better outcome, but given that PD-L1 expression is not correlated with HPV status, both HPV-positive and negative patients can be treated with combination immunotherapy and targeted therapies (27).

In the evolving era of molecular medicine, next-generation sequencing (NGS) genomic profiling assays can help to identify potentially targetable therapy options and can be especially valuable for cancers with limited effective treatment options. Both of the aforementioned patients underwent large panel NGS molecular testing, the findings discussed above. While the targetability of specific mutations was limited to the inactivating PTCH1 alteration in the first patient case, NGS can also provide information on additional biomarkers, such as TMB and microsatellite status that can also identify additional treatment options. Both TMB and microsatellite instability (MSI-high) are recognized as histology agnostic FDA approved biomarkers for pembrolizumab. While at this time our limited understanding of numerous genomic alterations precludes definitive treatment options for patients with these mutations, the further development of clinical trials will help provide more information, and the fact that these patients were treated with pembrolizumab with treatment response far exceeding the current standards of care is important in providing prognostic and therapeutic information in this patient population.

The evolving landscape of treatment of metastatic penile cancer is a subject of great research focus, with multiple clinical trials seeking to enroll patients with the aim to improve clinical outcomes. There was a phase II trial of pembrolizumab for advanced penile squamous cell carcinoma following prior systemic chemotherapy but unfortunately this was terminated due to poor patient accrual (NCT02837042), illustrating the challenge of assessing novel therapy options in patients with rare malignancies such as penile cancer and further supporting the value of case series evidence. Recent work by our group with a PSCC mouse model has demonstrated a potential role of combinatory strategies with immunotherapy and targeted therapies (29), these could lay the foundation for future clinical trials in refractory PSCC.

The lack of data for new approaches to treat metastatic penile cancer serves as a conundrum for clinicians with patients with advanced disease. While we realize our limited case series of two patients treated with pembrolizumab for their metastatic penile cancer does not provide a level of evidence to change treatment paradigms, the authors feel that it is of the utmost importance to report on these two patients with extraordinary responses and durable benefit from immunotherapy with pembrolizumab for their refractory metastatic disease. While penile carcinoma is a rare disease, it is still a significant cause of mortality, as it is responsible for 383 deaths yearly in North America and over 15,000 deaths yearly in the world (2). This report aims to highlight the importance of genomic testing in refractory PSCC and the potential clinical benefit of ICB in specific patients when clinical trials are not available. We look forward to future innovations in the field starting with basic science work to further improve our understanding of this disease, as well as multi-institutional collaborative efforts in clinical trials with support from industry and advocacy groups to improve the survival of patients with PSCC.

## Data Availability Statement

The original contributions presented in the study are included in the article/supplementary material. Further inquiries can be directed to the corresponding author.

## Ethics Statement

Written informed consent was obtained from the individual(s) for the publication of any potentially identifiable images or data included in this article.

## Author Contributions

All authors have approved this submission to Frontiers in Oncology. All persons listed as authors have contributed to preparing the manuscript and no person(s) other than the authors have contributed significantly to its preparation. All authors contributed to the article and approved the submitted version.

## Conflict of Interest

PS: NCCN bladder and penile cancer panel vice-chair, and President of the Global Society of Rare GU Tumors. CW: Consultant for the Molecular Tumor Boards of Intermountain Healthcare and Jackson Genetic Laboratories, PRN paid employee of HCA Mission Hospital. RJ: Advisory board—Pfizer, Seattle Genetics; Speakers bureau—Astellas/Seattle Genetics.

The remaining authors declare that the research was conducted in the absence of any commercial or financial relationships that could be construed as a potential conflict of interest.

The reviewer GS declared a past co-authorship with two of the authors JC, PS to the handling editor.
